# Pilot study to evaluate a novel measure of self-perceived competencies among dental students

**DOI:** 10.1186/s12909-022-03663-6

**Published:** 2022-07-29

**Authors:** Gerhard Schmalz, Henrike Kullmann, Tom Sensky, Deborah Kreher, Rainer Haak, Stefan Büchi, Dirk Ziebolz

**Affiliations:** 1 Department of Cariology, Endodontology and Periodontology, University of Leipzig, Liebigstr. 12, 04103 Leipzig, Germany; 2 Department of Medicine, Centre for Mental Health, Imperial College London, London, UK; 3 Clinic for Psychotherapy and Psychosomatics “Hohenegg”, Meilen, Switzerland

**Keywords:** Dental education, PRISM, Visual metaphor, Undergraduate education, Self-reflection, Communication

## Abstract

**Background:**

PRISM is a novel approach to support self-reflection and learning appraisal in dental students, based on a visual metaphor. The aim of this study was to evaluate whether PRISM measurments would be reproducible and sensitive to detect learning progress in undergraduate dental students in their clinical years.

**Methods:**

Voluntarily participating dental students were included. To evaluate reproducibility, a mixed cohort of 10 students each in 3^rd^, 4^th^ and 5^th^ year (total *n* = 30) was recruited and received three identically structured PRISM interviews within one week without any other teaching events. To assess perceived learning progress, 29 volunteer 3^rd^ year students participated in three interviews during their clinical simulation course (beginning, middle, end). Distances between Subject and Objects was measured in millimeter; objects were classified into close or branched clusters depending on their distance from each other on the PRISM board.

**Results:**

Values for perceived competencies within PRISM interviews during one week were comparable between the three time points in the mixed cohort (*n* = 30; *p* > 0.05). Comparing the three subgroups (3^rd^, 4^th^ and 5^th^ year, each *n* = 10), PRISM indicated that 3^rd^ year students perceived their competencies are significantly lower than the 4^th^ and 5^th^ year students (*p* < 0.01). 3^rd^ year students had less often a branched cluster of objects than the other two groups (*p* < 0.05).

PRISM showed that over time, 3^rd^ year students perceived a gain in their competencies in conservative dentistry and its sub-disciplines (*p* ≤ 0.01). The PRISM data indicated that by the end of the simulation course, the students appeared to show higher discrimination of their self-perceptions between sub-topics in conservative dentistry than at the start of the course (*p* = 0.01).

**Conclusion:**

PRISM yields a reproducible measure of individual students' learning progress. It is a promising novel approach for appraisal in dental education. Further work is needed to confirm the generalisability of the findings.

**Supplementary Information:**

The online version contains supplementary material available at 10.1186/s12909-022-03663-6.

## Background

The ability for appropriate self-assessment and reflection appear crucial in medical professionals but this core competency is limited across physicians [1]. It is therefore not surprising that self-reflection and reflective practice is an issue of high scientific relevance in medical education [2]. As a theoretical basis, different models of reflection exist, which illustrate reflective practice within two potential dimensions, i.e. an iterative dimension, whereby reflection is triggered by experience and a vertical dimension, which includes different levels of reflection on experience [2]. Against this theoretical background, many potential measures have been developed to assess the ability to self-reflect or self-assess. Williams et al. categorized them in a systematic review into three types, rubrics, self-reported scales and observed behavior [3]. These instruments need to be applied individually, depending on different influential factors, while no instrument appears superior to others [3]. Recently, further approaches to support and/or foster self-assessment and reflective practice have been reported, including virtual reality [4], briefing and debriefing sessions [5], or video-based approaches [6]. Those are only several examples from the emerging field of self-assessment and self-reflection in medical education research.

Recently, a novel tool has been introduced to support self-reflection and to facilitate learning progress. PRISM is a visual tool based on a metaphor [7]. The applications for PRISM are broad; while initially developed to visualize suffering, a growing variety of applications have been reported [8]. The structure of the PRISM task as a visual metaphor allows it to generate information about personally salient appraisals [8]. Thus, this method has also a potential in medical education. To the authors’ knowledge this is the first study to examine the use of PRISM in medical education.

The aims of this pilot observational study were to demonstrate the potential of PRISM as a tool to I) facilitate and II) quantify perceptions of learning progress in a small group of clinical dental students and to test the reliability of the measure. Based on these two aims, the study tested two hypotheses: I) repeated quantitiative measurements of PRISM are stable within one week without education events in a mixed cohort of undergraduate dental students. II) students’ PRISM responses will reflect expected improvements in competencies (including knowledge, skills and perceived training need) during a particular course.

## Methods

### Study design

This observational study has been reviewed and approved by the ethics committee of the Medical Faculty of University of Leipzig, Germany (No: 117/20-ek). All participants were informed verbally and in writing about the study and provided their written informed consent. The study consisted of two parts: (I) repeated application of PRISM in a cohort of 30 undergraduate dental students over one week without any education events and (II) the evaluation of PRISM findings during a clinical simulation course in conservative dentistry and periodontology.

### Participants

Volunteers were recruited among undergraduate dental students in their clinical years of study. For part (I), 10 students each from 3^rd^, 4^th^ and 5^th^ year of study were included. The 3^rd^ year students were recruited prior to their simulation course and 4^th^ as well as 5^th^ year students were recruited after their clinical course in conservative dentistry and periodontology. For part (II), 29 students in 3^rd^ year were included and followed-up during eight weeks of their clinical simulation course in conservative dentistry. Students were included irrespective of their age or gender. There were no further inclusion or exclusion criteria.

### PRISM interviews

**Fig. 1:**
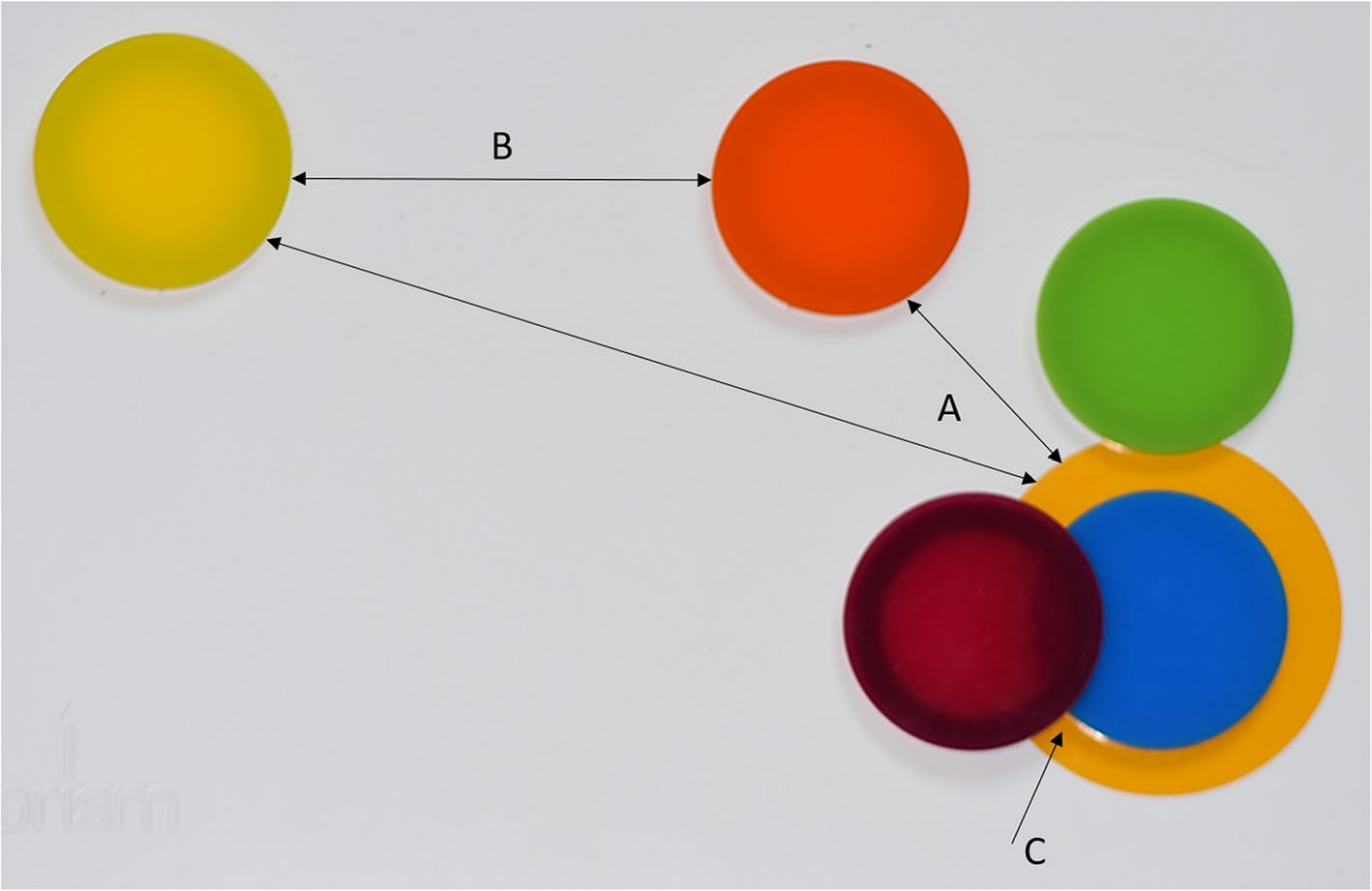
Measurments of the PRISM task. All distances were measured in millimetre using a ruler. **A**: Distance between the outer edge of Object and Subject disc were measured as the main quantitative result. **B**: Distance between different Objects can be measured and were in the current study only used to classify the cluster of Objects (see Fig. 2). **C**: If any Object disc touches the edge of the Subject disc, its distance was set as “0 mm” (as was the distance for Objects placed in the center of the Subject)

**Fig. 2:**
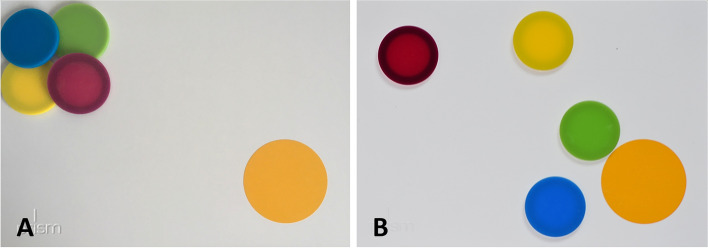
Illustration of Object clustering. **A** Objects are close to each other (“close cluster”), **B** Objects are branched with a remarkable distance between each other (“branched cluster”)

PRISM was initially developed in the field of psychology/psychosomatic medicine to assess and visualize patient suffering [7]. For the PRISM task, a white metal board (297 × 210 mm), represents a defined context (in this study, ‘your dental studies’). The board includes a fixed yellow circle (7 cm in diameter) at the bottom right hand corner representing the identified Subject (in this study, ‘myself as a X-year dental student’). Different coloured Object discs (5 cm diameter) can then be placed in relation to the Subject. The Object discs represent different study-related issues, e.g. “your theoretical skills in the field of conservative dentistry”. For quantitiative assessment, the distance between Subject and Object was measured in millimeters (Fig. 1). Beside of the total distance between Subject and Object, the relationship between Objects was also assessed according to the distance between them and whether different Objects were placed in a close or branched cluster (Fig. 2). This clustering was assessed to evaluate the perceived relationship between the different sub-topics of the same topic (disciplines of conservative dentistry). This allowed assessment of the ways in which students distinguish between the sub-topics and whether their perceptions change during the course. For example, placing sub-topics into a close cluster was taken to indicate a low distinction between the topics, what might be caused by limited experiences and knowledge in this respect. Placing topics in a branched cluster was taken to indicate a high distribution between topics, potentially related to a gain in experiences in the different sub-topics.

### PRISM interviews

One dentist (GS), trained in the use of PRISM, performed all the interviews. This dentist was not involved in the general appraisal of the clinical courses of the students. The PRISM task was used in addition to and independently of students’ regular appraisals. Interview settings were standardized as far as was possible. Interviews started with an explanation of the task and one example question (“Imagine this blue disk is burger and this green disc is broccoli. Where would you put these disks to reflect how much do you like them at the moment?”). A short explanation was repeated at each interview appointment. Interviews consisted of 18 questions, regarding students; skills, knowledge and their perception of remaining training needs in different fields in dentistry (Table S1). The task aimed to evaluate students’ subjectively perceived competencies, i.e. their recent self-perceived knowledge, practical skills and related remaining training needs. In part (I), all participants underwent three interviews within one week (Monday – T1, Wednesday – T2, Friday – T3), including the same questions (each 10–12 min). For part (II), students were interviewed at the beginning, middle (after four weeks) and end of their simulation course in conservative dentistry. Each interview lasted 10–12 min.

### Statistical analysis

The statistical analysis was performed with SPSS for Windows, version 24.0 (SPSS Inc., U.S.A.). Values are presented as mean values with standard deviation or percentage, respectively. For part (I), values between the three time points were compared to each other for the total cohort and also in the three sub-groups (3^rd^, 4^th^ and 5^th^ year). For part (II), values for the total cohort were compared between beginning, middle and end of the simulation course. Normal distribution was tested with Shapiro–Wilk test. Based on normality of distribution, the general linear model or Friedman test was applied, while sign test was used for ordinal data. More than two independent, non-normal distributed samples were compared using Kruskal–Wallis test. For the analysis of categorical data, a chi-square test was applied. The significance level was set at *p* < 0.05.

## Results

### Participants

For Part (I), 30 students were included, which had a mean age of 23.9 ± 3.3 years and a balanced gender distribution (50% male and female, respectively). In this group, 10 students each were in 3^rd^, 4^th^ and 5^th^ study year. The sample for Part (II) consisted of 3^rd^ year students (*n* = 29) undergoing a course in conservative dentistry. They had a mean age of 22.9 ± 3.0 years and had a majority of female participants (72% vs. 28%).

### Evaluation of PRISM after 3 interviews

When PRISM was repeated at three times within one week (Monday, Wednesday, Friday), the variation within the whole group (*N* = 30) between values did not reach statistical significance (*p* > 0.05, Table 1). Among the three subgroups, 4^th^ and 5^th^ year dental students showed stable values across the week (*p* > 0.05, Table 2). Within the 3^rd^ year students, significant differences were found between the three measurement points (*p* < 0.05) despite the students having had no educational events during the week, while only the clusters remained stable (Table 2).

**Table 1:** PRISM results for the total cohort (*n* = 30) including students from 3^rd^, 4^th^ and 5^th^ year (each *n* = 10) at three time points within one week (Monday, Wednesday, Friday). Values are given as mean and standard deviation and represent values in millimetre. Significance level was *p* < 0.05

		**T1**	**T2**	**T3**	***p*-value**
Theoretical knowledge	Whole field of conservative dentistry	67.6 ± 64.2	66.3 ± 64.2	70.5 ± 60.5	0.12
	Cariology	65.8 ± 65.9	60.5 ± 57.2	66.7 ± 56.5	0.50
	Endodontology	87.6 ± 77.9	81.1 ± 71.1	83.73 ± 67.2	0.83
	Periodontology	72.1 ± 78.6	59.3 ± 70.6	59.7 ± 65.3	0.22
	Restorative dentistry	74.5 ± 68.8	68.7 ± 62.3	71.1 ± 57.2	0.59
	Prevention	57.1 ± 70.0	47.13 ± 66.7	51.9 ± 64.5	0.06
	Cluster knowledge	-	-	-	0.45
Practical skills	Whole field of conservative dentistry	75.0 ± 77.3	72.7 ± 64.1	73.7 ± 61.2	0.99
	Cariology	77.03 ± 84.7	72.6 ± 71.5	69.0 ± 67.9	0.39
	Endodontology	107.3 ± 71.0	104.1 ± 64.4	102.3 ± 59.6	0.76
	Periodontology	79.4 ± 83.6	73.9 ± 76.5	70.9 ± 75.2	0.14
	Restorative dentistry	86.8 ± 80.5	85.7 ± 74.2	81.2 ± 67.9	0.40
	Prevention	69.5 ± 89.0	58.3 ± 71.2	56.9 ± 70.4	0.92
	Cluster skills	-	-	-	0.13
Training need	Whole field of conservative dentistry	89.2 ± 70.3	89.1 ± 61.5	88.6 ± 66.0	0.89
	Cariology	91.0 ± 72.1	80.2 ± 66.1	75.6 ± 64.4	0.14
	Endodontology	111.3 ± 69.0	109.6 ± 60.0	111.4 ± 65.3	0.97
	Periodontology	86.6 ± 79.0	80.3 ± 74.7	81.9 ± 75.4	0.55
	Restorative dentistry	100.3 ± 81.0	102.2 ± 68.6	92.8 ± 67.5	0.22
	Prevention	76.6 ± 80.1	64.6 ± 76.3	60.8 ± 72.7	0.06
	Cluster training need	-	-	-	0.23

**Table 2:** P-values for the comparison of PRISM values between T1-T3 within students from 3^rd^, 4^th^ and 5^th^ year (each *n* = 10). Significance level was *p* < 0.05

		**3**^**rd**^** year (*n* = 10)**	**4**^**th**^** year (*n* = 10)**	**5**^**th**^** year (*n* = 10)**
Theoretical knowledge	Whole field of conservative dentistry	0.79	0.12	0.11
	Cariology	0.29	0.59	0.07
	Endodontology	**0.02**	0.21	0.15
	Periodontology	**0.02**	0.06	0.51
	Restorative dentistry	**0.04**	0.30	0.07
	Prevention	0.07	0.15	0.19
	Cluster knowledge	0.99	0.63	0.99
Practical skills	Whole field of conservative dentistry	0.17	0.20	0.92
	Cariology	** < 0.01**	0.72	0.23
	Endodontology	**0.01**	0.61	0.07
	Periodontology	**0.01**	0.82	0.74
	Restorative dentistry	** < 0.01**	0.14	0.46
	Prevention	**0.01**	0.42	0.34
	Cluster skills	0.99	0.50	0.63
Training need	Whole field of conservative dentistry	0.59	0.58	0.25
	Cariology	**0.04**	0.84	0.93
	Endodontology	0.19	0.50	0.67
	Periodontology	0.11	0.41	0.81
	Restorative dentistry	**0.02**	0.15	0.24
	Prevention	0.14	0.31	0.77
	Cluster training need	0.50	0.50	0.13

Comparing the three subgroups, i.e. 3^rd^, 4^th^ and 5^th^ year students (each *n* = 10), 3^rd^ year students had significantly higher values in theoretical knowledge, practical skills and training need, including the whole field of conservative dentistry as well as all sub-topics (*p* < 0.01, Table 3). This indicates that 3^rd^ year students perceived their theoretical knowledge and practical skills as less well developed and their training needs as greater. Also, 3^rd^ year students showed a lower amount of branched cluster of objects than the other two groups (*p* < 0.05, Table 3).

**Table 3:** Comparison of PRISM data between students from 3^rd^, 4^th^ and 5^th^ year (each n = 10) at the third PRISM interview within one week (T3). Values are given as mean and standard deviation and represent values in millimetre. Significance level was *p* < 0.05

		**3**^**rd**^** year (*n* = 10)**	**4**^**th**^** year (*n* = 10)**	**5**^**th**^** year (*n* = 10)**	***p*-value**
Theoretical knowledge	Whole field of conservative dentistry	143.4 ± 50.3	35.3 ± 12.5	32.8 ± 15.6	** < 0.01**
	Cariology	124.6 ± 43.2	28.7 ± 39.9	46.9 ± 32.6	** < 0.01**
	Endodontology	167.9 ± 27.3	31.7 ± 17.2	51.6 ± 38.3	** < 0.01**
	Periodontology	140.7 ± 47.7	26.9 ± 17.6	11.5 ± 9.3	** < 0.01**
	Restorative dentistry	126.4 ± 50.4	56.5 ± 45.7	30.4 ± 20.9	** < 0.01**
	Prevention	132.1 ± 47.3	14.7 ± 19.5	9.0 ± 7.4	** < 0.01**
	Cluster knowledge branched	20%	60%	80%	**0.02**
Practical skills	Whole field of conservative dentistry	145.0 ± 46.1	38.5 ± 23.1	37.7 ± 30.7	** < 0.01**
	Cariology	156.3 ± 40.7	19.7 ± 24.2	30.9 ± 27.2	** < 0.01**
	Endodontology	162.6 ± 41.6	60.5 ± 27.1	85.2 ± 51.5	** < 0.01**
	Periodontology	167.3 ± 40.8	34.4 ± 25.5	11.0 ± 10.3	** < 0.01**
	Restorative dentistry	157.6 ± 38.7	49.6 ± 51.7	36.5 ± 29.2	** < 0.01**
	Prevention	144.5 ± 46.0	17.9 ± 31.4	8.3 ± 6.1	** < 0.01**
	Cluster skills branched	10%	90%	90%	** < 0.01**
Training need	Whole field of conservative dentistry	164.0 ± 49.3	58.8 ± 29.8	43.1 ± 33.3	** < 0.01**
	Cariology	154.6 ± 40.3	36.1 ± 27.6	36.1 ± 24.4	** < 0.01**
	Endodontology	184.3 ± 24.6	65.6 ± 42.6	84.3 ± 47.5	** < 0.01**
	Periodontology	171.8 ± 40.7	55.5 ± 44.5	18.3 ± 20.4	** < 0.01**
	Restorative dentistry	164.8 ± 34.6	75.3 ± 58.3	38.4 ± 26.3	** < 0.01**
	Prevention	151.3 ± 48.8	17.3 ± 27.7	13.7 ± 14.7	** < 0.01**
	Cluster training need branched	0%	90%	90%	** < 0.01**

### Evaluation of PRISM over 8 weeks of dental education

During the 3^rd^ year stusents’ simulation course, the PRISM task showed significant reductions in the distances between the Subject and Objects reflecting conservative dentistry or its sub-topics (*p* ≤ 0.01, Table 4). This indicated that over the duration of the simulation course, students perceived improvements in their skills and knowledge as well as reduction of their training needs. There was also a substantial change in clusters during the course – there were more branched clusters at the end of the course than at the beginning across the sub-topics regarding their practical skills (*p* < 0.01) and training need (*p* = 0.01; Table 4). This indicates that students at the end of the course distinguished between the sub-topics, potentially as a result of increased experiences and knowledge in those topics.

**Table 4:** PRISM data for the total 3^rd^ year cohort (*n* = 29) at three time points during the course of conservative dentistry (beginning, middle (after 4 weeks), end of the course (after 8 weeks)). Values are given as mean and standard deviation and represent values in millimetre. Significance level was *p* < 0.05

		**Beginning**	**Middle**	**End**	***p*-value**
Theoretical knowledge	Whole field of conservative dentistry	105.1 ± 58.6	74.6 ± 53.3	56.6 ± 43.0	** < 0.01**
	Cariology	89.3 ± 49.4	61.3 ± 49.1	62.3 ± 44.9	**0.01**
	Endodontology	156.2 ± 45.8	76.0 ± 49.3	60.0 ± 39.3	** < 0.01**
	Periodontology	134.7 ± 54.4	116.6 ± 59.4	66.3 ± 51.2	** < 0.01**
	Restorative dentistry	97.4 ± 55.1	74.8 ± 49.5	85.2 ± 44.8	**0.01**
	Prevention	97.4 ± 58.9	91.9 ± 56.4	74.0 ± 60.6	**0.01**
	Cluster knowledge	-	-	-	0.18
Practical skills	Whole field of conservative dentistry	120.1 ± 64.1	87.2 ± 49.8	75.9 ± 42.1	** < 0.01**
	Cariology	137.0 ± 56.7	92.7 ± 53.6	80.6 ± 51.9	** < 0.01**
	Endodontology	169.6 ± 42.7	158.6 ± 55.0	73.1 ± 44.2	** < 0.01**
	Periodontology	162.8 ± 45.4	155.0 ± 53.3	128.6 ± 67.4	**0.01**
	Restorative dentistry	135.7 ± 59.6	107.7 ± 52.9	92.3 ± 46.6	** < 0.01**
	Prevention	132.8 ± 56.9	122.0 ± 64.8	107.0 ± 66.5	**0.01**
	Cluster skills	-	-	-	** < 0.01**
Training need	Whole field of conservative dentistry	150.8 ± 56.2	125.6 ± 54.5	97.1 ± 47.3	** < 0.01**
	Cariology	148.7 ± 49.8	110.2 ± 51.2	95.4 ± 47.0	** < 0.01**
	Endodontology	184.9 ± 28.1	156.1 ± 34.2	95.6 ± 47.2	** < 0.01**
	Periodontology	168.8 ± 37.6	161.3 ± 42.6	130.8 ± 42.7	** < 0.01**
	Restorative dentistry	153.5 ± 48.4	125.0 ± 48.7	124.8 ± 48.0	** < 0.01**
	Prevention	141.7 ± 57.7	120.7 ± 57.4	104.1 ± 52.2	** < 0.01**
	Cluster training need	-	-	-	**0.01**

## Discussion

### Main results

In a mixed cohort of undergraduate dental students in their clinical years, PRISM values remained stable over one week in the absence of any education events. This supports the reproducibility of the PRISM task i.e. its reliability. Over the duration of a clinical simulation course, PRISM indicated that students’ perceived competencies improved, indicating that PRISM is sensitive to visualize self-perceived learning progress.

To the authors’ knowledge, this is the first quantitative assessment of PRISM in medical or dental education, or indeed any higher education. There is therefore no directly comparable published work. As noted in the introduction, PRISM is a visual metaphor initially applied to measure suffering [9] but its use has extended more recently to a growing range of different applications [8]. In its original application, the PRISM task has been demonstrated to have good reliability, yielding consistent results when repeated at intervals of a few hours to three days [9–11]. Further studies confirmed the reliability of PRISM to assess the disease burden of patients, e.g. for patients with psoriasis [12], and even for measuring pain-related suffering across different countries [13]. The present results of part (I) of the current study are consistent with this, indicating the PRISM yields reliable data in the setting of dental education.

In this context, it was conspicious that 3^rd^ year students showed less stable results in part (I) than the 4^th^ and 5^th^ year students. This might be explained by the structure of dental studies in Germany. In the first two years of dental studies, students focus on basic subjects in medicine and dental technology. In their 3^rd^ year, students encounter conservative dentistry and its sub-topics for the first time. Students might have difficulty understanding their competencies in a subject they have never heard about, or may even mix up the sub-topics across interviews. This would result in the limited reproducibility in this particular year group. On the other hand, one could argue that students should perceive themselves as incompetent in a subject in which the students had no experience or teaching. On this basis, PRISM values would be expected to be particularly stable in this year group. Another possibility is that, as expected, the PRISM task encourages self-reflection by the students and they attempt to guage to what extent their learning to date will be relevant to topics they have not yet encountered. Further work is needed to resolve this.

Quantitative analysis for PRISM has been repeatedly performed in its original application form; different clinical studies have measured the suffering or disease burden of patients, e.g. with ulcerative colitis, chronic inflammatory vulvar disease, liver cirrhosis or organ transplantation [14–17]. These studies choosed different ways of quantification, including measurement in centimentre, millimeter or distinction of values [14–17]. Based on the individuality of the PRISM task, a remarkable range is often observed, especially if values are measured in millimeter, however, this also brings a high sensitivity [15].

A reduction of the distance between Subject and Objects was observerd during the clinical simulation course, reflecting improvements in students’ perceived competencies. This supports PRISM as a sensitive tool to visualize and measure learning progress in undergraduate dental students. As expected, the largest difference was between 3^rd^ year and the two other year groups (Table 3). This can be explained by the fact that 3^rd^ year students undergo the simulation course, while 4^th^ and 5^th^ year students work with real patients. It is recognised that pre-clinical simulation in dental education is not entirely satisfactory, because simulated and real patients differ significantly [18]. Considering the simulation course alone, 3^rd^ year students perceive a substantial improvement in their competencies during the course (Table 4).

The clustering of Objects i.e. students’ perceived competencies yielded further information about the students’ perceptions. At the beginning of the 3^rd^ year simulation course, the students placed all the sub-topics in a tight cluster, consistent with a lack of discrimination, expected because of their lack of prior experience of the sub-topics. As the course progressed, the sub-topics showed a branched structure, consistent with the students developing a more differentiated and nuanced appraisal of the sub-topics due to their teaching and their growing practical experience. As expected, the branched structure of sub-topics was also evident in the responses of the 4^th^ and 5^th^ year students. The clustering of PRISM Objects might therefore provide a visual summary of the student’s conceptualization of a domain of knowledge. How, using PRISM, a student clusters the sub-topics of one overall topic or domain e.g. the whole field of conservative dentistry, may provide insights into how the students perceives structural relationships and hierarchies among topics or sets of technical skills, and into the student’s capacity to distinguish among different components of knowledge or technical performance. This is also expected to substantially facilitate discussion between student and teacher not only because PRISM provides a visual summary as the basis for discussion but also because of the properties that PRISM shares with other metaphors [8]. For these reasons, further work on clustering of Objects in the PRISM task in medical or dental education settings might be fruitful.

This pilot study has shown that PRISM can be applied reliably to assess quantitatively students’ perceptions of their learning needs and is sensitive to change. Further work is needed to assess how PRISM compares with existing numerical scales and other appraisal tools used in dental education. Numerical and similar scales are convenient for students and teachers because they are simple to administer and evaluate [19]. PRISM, although brief to administer, takes longer to administer and evaluate that a umerical scale. However, PRISM has the advantage that it can be readily incorporated into the appraisal discussion between student and teacher, as has been shown in others of its applications [8].

*Strengths and limitations*: The main limitation of the present work is that it is a pilot study, involving a small number of selected students and a single teacher. Comparisons between the year groups were limited because of their small numbers. The students were volunteers and might thus have been particularly motivated to work with PRISM. The teacher was well trained and experienced in the use of PRISM and some training will be necessary for other teachers applying PRISM. Also, without knowing whether the PRISM interviews were likely to work, it would have been wrong to integrate them into the students’ course, but this meant that the PRISM interviews were separate from and additional to the course. However, the results indicate the feasibility of applying PRISM to dental education. Nevertheless, further work is necessary to confirm the generalizability of the findings. As noted above, there is no gold standard for student self-appraisal, but further work should at least compare PRISM with other self-appraisal tools.

The current study has several key strengths. It evaluated a novel tool for self-assessment of competencies and learning needs in undergraduate dental students. Although the PRISM measure has been applied in a variety of settings, to the authors’ knowledge this is the first study in which PRISM has been applied in higher education. It is also the first study in which interpretation of PRISM has been extended to include clustering of the objects under investigation. The results of the study have been promising – even with the small numbers of students recruited, the PRISM task has been shown to be reliable and to yield results consistent with expectations.

## Conclusion

This pilot study has demonstrated that PRISM has potential as a reliable tool to quantify perceived competencies in undergraduate dental students in their clinical years of study. Furthermore, PRISM generates a visual summary of each student’s perceived learning progress. Even allowing for the limitations of the study, its results suggest that use of PRISM would be a promising approach to extend the instruments for appraisal in dental education. If the generalizability of PRISM is confirmed among dental students, it should also be applicable to medical students as well as to those following postgraduate studies.

## Supplementary Information

**Additional file 1: Table S1**: The questions within the PRISM task in thecurrent study.

## Data Availability

The datasets used and/or analysed during the current study are available from the corresponding author on reasonable request. The data are not publically available, because of the psedonymisation and data protection guidelines according to the ethics approval.

## References

[1] Davis DA, Mazmanian PE, Fordis M, Van Harrison R, Thorpe KE, Perrier L (2006). Accuracy of physician self-assessment compared with observed measures of competence: a systematic review. JAMA.

[2] Mann K, Gordon J, MacLeod A (2009). Reflection and reflective practice in health professions education: a systematic review. Adv Health Sci Educ Theory Pract.

[3] Williams JC, Ireland T, Warman S, Cake MA, Dymock D, Fowler E, Baillie S (2019). Instruments to measure the ability to self-reflect: A systematic review of evidence from workplace and educational settings including health care. Eur J Dent Educ.

[4] Wu JH, Du JK, Lee CY (2021). Development and questionnaire-based evaluation of virtual dental clinic: a serious game for training dental students. Med Educ Online.

[5] Botelho M, Bhuyan SY (2021). Reflection before and after clinical practice-Enhancing and broadening experience through self-, peer- and teacher-guided learning. Eur J Dent Educ.

[6] Krause F, Ziebolz D, Rockenbauch K, Haak R, Schmalz G. A video- and feedback-based approach to teaching communication skills in undergraduate clinical dental education: The student perspective. Eur J Dent Educ. 2021. 10.1111/eje.12682. Epub ahead of print. 10.1111/eje.12682 33728768

[7] Büchi S, Sensky T, Sharpe L, Timberlake N (1998). Graphic representation of illness: a novel method of measuring patients' perceptions of the impact of illness. Psychother Psychosom.

[8] Sensky T, Büchi S (2016). PRISM, a Novel Visual Metaphor Measuring Personally Salient Appraisals, Attitudes and Decision-Making: Qualitative Evidence Synthesis. PLoS ONE.

[9] Büchi S, Buddeberg C, Klaghofer R, Russi EW, Brändli O, Schlösser C, Stoll T, Villiger PM, Sensky T (2002). Preliminary validation of PRISM (Pictorial Representation of Illness and Self Measure) - a brief method to assess suffering. Psychother Psychosom.

[10] Lima-Verde AC, Pozza DH, Rodrigues LL, Velly AM, Guimarães AS (2013). Cross-cultural adaptation and validation for Portuguese (Brazilian) of the pictorial representation of illness and self measure instrument in orofacial pain patients. J Orofac Pain.

[11] Kassardjian CD, Gardner-Nix J, Dupak K, Barbati J, Lam-McCullock J (2008). Validating PRISM (Pictorial Representation of Illness and Self Measure) as a measure of suffering in chronic non-cancer pain patients. J Pain.

[12] Fotiou K, Hofmann M, Kaufmann R, Thaci D (2015). Pictorial representation of illness and self measure (PRISM): an effective tool to assess the burden of psoriasis. J Eur Acad Dermatol Venereol.

[13] Brady B, Veljanova I, Andary T, Southwell T, Chipchase L (2019). Recognising ethnocultural diversity in chronic pain assessment: validation of the Pictorial Representation of Illness and Self Measure (PRISM) for use with culturally diverse communities. Health Qual Life Outcomes.

[14] Ghosh S, Sensky T, Casellas F, Rioux LC, Ahmad T, Márquez JR, Vanasek T, Gubonina I, Sezgin O, Ardizzone S, Kligys K, Petersson J, Suzuki Y, Peyrin-Biroulet L (2020). A Global, Prospective, Observational Study Measuring Disease Burden and Suffering in Patients with Ulcerative Colitis Using the Pictorial Representation of Illness and Self-Measure Tool. J Crohns Colitis.

[15] Corazza M, Virgili A, Toni G, Valpiani G, Morotti C, Borghi A (2020). Pictorial Representation of Illness and Self-Measure to assess the perceived burden in patients with chronic inflammatory vulvar diseases: an observational study. J Eur Acad Dermatol Venereol.

[16] Kabar I, Hüsing-Kabar A, Maschmeier M, Völler C, Dümke M, Schmidt HH, Heinzow H (2018). Pictorial Representation of Illness and Self Measure (PRISM): A Novel Visual Instrument to Quantify Suffering in Liver Cirrhosis Patients and Liver Transplant Recipients. Ann Transplant.

[17] Goetzmann L, Seiler A, Benden C, Boehler A, Büchi S, Jenewein J, Ruettner B, Mueller-Alcazar A, Weierstall R (2018). Transplantation experience as a predictor for quality of life during the first 6 months after lung transplantation. Clin Transplant.

[18] Tanzawa T, Futaki K, Tani C, Hasegawa T, Yamamoto M, Miyazaki T, Maki K (2012). Introduction of a robot patient into dental education. Eur J Dent Educ.

[19] O'Donnell JA, Oakley M, Haney S, O'Neill PN, Taylor D (2011). Rubrics 101: a primer for rubric development in dental education. J Dent Educ.

