# Supplementary material for: Pilot study to evaluate a novel measure of self-perceived competencies among dental students
Source: BMC Med Educ. 2022 Jul 29;22:582. doi: 10.1186/s12909-022-03663-6 (PMC9336052; doi:10.1186/s12909-022-03663-6)
Supplement: Supplementary file 1 — Additional file 1: Table S1: The questions within the PRISM task in thecurrent study. [file 12909_2022_3663_MOESM1_ESM.docx]

**Supplementary tables**

**Table S1**: The questions within the PRISM task in the current study.

|  | |
| --- | --- |
| Theoretical knowledge | How do you perceive your theoretical knowledge in the whole field of conservative dentistry? |
|  | How do you perceive your theoretical knowledge in Cariology? |
|  | How do you perceive your theoretical knowledge in Endodontology? |
|  | How do you perceive your theoretical knowledge in Periodontology? |
|  | How do you perceive your theoretical knowledge in Restorative dentistry? |
|  | How do you perceive your theoretical knowledge in Prevention? |
| Practical skills | How do you perceive your practical skills in the whole field of conservative dentistry? |
|  | How do you perceive your practical skills in Cariology? |
|  | How do you perceive your practical skills in Endodontology? |
|  | How do you perceive your practical skills in Periodontology? |
|  | How do you perceive your practical skills in Restorative dentistry? |
|  | How do you perceive your practical skills in Prevention? |
| Training need | How much training need to you perceive in the whole field of conservative dentistry? |
|  | How much training need to you perceive in Cariology? |
|  | How much training need to you perceive in Endodontology? |
|  | How much training need to you perceive in Periodontology? |
|  | How much training need to you perceive in Restorative dentistry? |
|  | How much training need to you perceive in Prevention? |
